# Empyema

**Published:** 1865-10

**Authors:** Jas. S. Whitmire

**Affiliations:** Metamora, Illinois


CHICAGO

MEDICAL JOURNAL.

VOL. XXII.] OCTOBER, 1865. [No. 10.

### ORIGINAL COMMUNICATIONS.

### EMPYEMA.

By JAS. S. WHITMIRE, M. D. of Metamora, Illinois.

In the course of four years, it has been my fortune to have under my care four cases of Empyema, all of which have perfectly recovered, and the persons are now enjoying as good health as was ever allotted to either, previous to having the disease.

Therefore, without going into any general treatise on the disease, the theory of which is fully ventillated in our standard works, I propose, for the timid country practitioner, to give a brief history of three of the cases and their treatment. This, I do under the firm conviction that very many go down to an untimely grave from either ignorance or timidity, under the misnomer of Phthisis, Chronic Hepatitis, Hydrothorax, Cardiac disease, &c., &c. Now if this class of good men could be aroused to a just sense of their duty, and the dread of an operation be lessened in their minds, very many valuable lives might be annually saved; the profession elevated, and physicians respected in country practice, according to their real merit.

The first case to which my attention was called was in the family of David Watson, a few miles from this place. I was attending Mrs. Watson in case of acute rheumatism, March 1st, 1860, at the time, and she was not getting along just as well as I could have desired, so that the family had become quite impatient. During this period of impatience, her son Edwin had an attack of what the doctor (an eclectic) called, an Intermittent Billious Fever. The young man was 21 years of age, and, on account of the slow recovery of his mother, exercised his undoubted prerogative of employing whomsoever he pleased, and consequently sent for another physician to attend him. I supposed at the time that the Dr. made his diagnosis, as he did, from the boy having had a chill, no attention having been paid to the physical signs. When I arrived the next day on my usual visit, I discovered another patient in the opposite corner of the same room. On inquiry, I found that another physician had been sent for. I asked, what the Dr. had called the disease: How the boy was attacked; how he had been since held, and many other questions partly from curiosity, and partly that I might observe more closely the progress of the disease. I was asked by his father to examine him and give my opinion for his own satisfaction, which I did. I found that he had been attacked with Pleuro-pneumonia of the middle lobe of the right lung, and so stated the fact, and in connection the danger his son was in, and that, if he did not wish to employ me, he had better send for another physician. He did not, however, heed the advice, and therefore, the eclectic and I each attended our own patient. I, however, kept a steady watch over the eclectic's patient as well as my own; the Dr. assuring the family from day to day that Edwin was better which I never contradicted in the least, but with my closest scrutiny "*I could not see it.*" In the course of two weeks I discharged my patient as convalescent, and left my eclectic friend "*alone in his glory*"—and in my opinion, exceedingly well pleased at getting rid of my presence. Still harping on the speedy recovery of his charge.

Edwin was taken sick about the first of March, 1860, and after the middle of that month I did not see him, till near the middle of April. I found him then, the next day after an operation had been performed by Dr. Wilson of Washington. The Dr. on his return, called upon me, by consent of the family, to take charge of the patient as the distance was too great for him to pay that attention to the case, as necessity required. He informed me at the same time that he had drawn off seven pints of puss. The operation of paracentesis had been performed between the 5th and 6th ribs and back of the nipple. Very much emaciated, Hectic fever, Colliquiative sweats, and a persistent diarrhœa. I found it difficult to draw the puss from the opening made, I therefore took a common female silver catheter to a silver-smith, had the end cut off, flattened for two inches and the end closed and rounded nicely and an opening made on one of the flat sides, the diameter of which was equal to that of the instrument. This I could slide in between the ribs very easily, producing but little pain and the pus would pass freely through it so that I had little difficulty in emptying the chest, I put him on Mur. Tinct. of Iron, Alum and Quinine, and ordered him rich animal broths, and if he wished it broiled beef or any kind of game.

Notwithstanding this I found the secretion of pus rather on the increase than otherwise and secreted in large quantities, so that my patient was fast succombing to this enormous drain upon the system. I kept a tent in the orifice, and drew off the pus every day that I might take note of the amount. Many days I would take a pint, others not more than half pint, always rinsing out the cavity with warm water by means of a gum cather which I would introduce to the botton of the cavity and inject through the open end with a small pointed glass syringe. Finding that some measure must be adopted to put a stop to this discharge, I determined to use a solution of Iodine after each rinsing with warm water. This I made of the following strength: ℞ Pot. Iod. gr. x, Tinct. Iod. ʒ ij, Aqua ℥ iv. This I would again draw off and introduce the tent. In the course of a week I found that the puss was materially diminished. I then added one more drachm of the tincture to the solution, and kept up the process for another week, at the end of which, seeing no evil consequences arise from its use, but rather, a slow, but steady improvement; I determined to only use the solution of Iodine every other day, and then only two ounces which I left remaining in the cavity; every day however I kept up the evacuating and rinsing processes. At the end of a few weeks the progress of the cure began to be very manifest in the lessening of the size of the cavity, either from adhesions or the resumption of the lungs of their normal functions by the permeability of the heretofore closed air cells, or perhaps both. I now lessened the strength of the solution and also its quantity using only one ounce and lengthened the number of days between its use. His appetite continued to improve; his strength and flesh increased, and when I could introduce but half an ounce of liquids into the cavity I closed the orifice; the wound healed kindly, and on the 15th day of August, 1860, I discharged my patient as cured and had the satisfaction of seeing him walking the streets in a short time without the assistance of a cane. The cavity, I have no doubt, closed by granulation of the two pleural surfaces and the chest is very little contracted, but the ribs do not move so freely in respiration as on the left side. At this time, four years since his recovery, there is little or no difference in the resonance on percussion of the two sides, and the respiratory murmur seems entirely healthy; and for the last three years he has worked steadily on a farm.

Case 2—Was a soldier belonging to one of the Indiana regiments. I do not recollect his name—I found him in one of the wards of the post hospital, at Smithland Ky., on the 2d of March, when I was put in charge. He told me that he had been taken two months previously and that he was at once sent to hospital. I found by reference to the hospital register that his disease had been diagnosed Pleuro-Pneumonia of right side. On measurement found that side two inches larger than the left; little or no movement of the ribs of that side during respiration, which was much hurried. Distinct bronchial, but no distinguishable vessicular respiration; flatness on percussion two inches above the nipple from spine to sternum and down to the base of the lung; frequent, small, soft pulse; hectic fever; colliquitive sweats; persistent diarrhœa and œdema of the feet and legs. On the 5th I opened the chest with a common bistory between the 5th and 6th ribs below and behind the nipple, and drew off two pints or more of thin purulent matter of a greenish yellow color. As in the former case I washed the cavity with warm water, but this time I lost no time in injecting the cavity with a solution of Iodine, in a similar manner as in Case 1. Every day for several days I evacuated the cavity by simply removing the tent and received from one to two gills of purulent matter each time. Finding the secretion of pus was not diminishing as fast as I thought it ought, on every alternate day I used instead of the solution of Iodine, a solution of Sulph. Zinc gr. v to the ounce of water to rinse out the cavity, and on the intermediate day I used the solution of Iodine leaving two ounces in the cavity. By the last of March very little pus escaped and the cavity was nearly closed; and as soon as I could hear tolerably distinctly the respiratory murmur, I closed the orifice which in a few days healed kindly, and my patient was able to sit up. At this time my hospital was broken up, and my patient removed to Paducah, Ky., where I lost sight of him till during the siege of Vicksburg, over fifteen months, where he found my regiment, and looked me up. He looked stout and rugged and told me that he had been on during the last six months, and had participated in all the battles previous to our investment and during the siege of that place. This man's constitutional and general treatment was substantially the same, as case 1. His recovery was more speedy and his health equally as good. One reason, probably, of his more speedy recovery was, that the compression of his lung was not so great and the Pneumonia had probably, a more effectual treatment.

Case 3.—Geo. W. Fawser, aged 13 years, was attacked February 15th, 1864 with Pleuro-pneumonia. On the 18th as I was passing the house, his father called me in; I represented to the parents the facts in the case, and made the usual prescription in such cases, and told them that the boy should have regular medic l attendance, and that if he did not get it the consequences might be serious. The parents however thought otherwise and paid me for the call, telling me at the same time that if the boy did not soon begin to recover, they would send for me. On the 26th I was passing, and again called in and found that there was already effusion of water or pus in the left pleural cavity there being dullness on percussiou and bronchial respiration, with absence of the respiratory murmer. I directed Hydrarg. Sub. Mur. and Pulv. Scilla, every four hours and after six powders had been taken senna and salts to produce free purgation. After this a large blister was to be applied all over the left chest. They again paid me for my call and said they would let me know from time to time how he was doing.

March 1st, the father called on me for a prescription and said his son was very much improved and that if he now had something to brace him up, he thought his son would soon recover. I thought very differently however, and so informed him of my doubts in the case. During the months of March and April I was called on, at my office, to prescribe several times for the boy, the father reporting him from time to time slowly improving. On the 7th of May I was called to see him. I found the child bolstered up in a chair looking almost bloodless, much emaciated, continued diarrhœa, night sweats, hectic fever, with respiration as hurried as a church mouse, on a hot day, and unable to walk across the floor. I stripped him, and it required no measurement, or other examination to diagnose the difficulty, the left breast being very much larger than the right; the heart was beating on the right side so that the right lung was also suffering from the enormous quantity of fluid accumulated in the left. I had no doubt from all the circumstances, that it was pus, and that the boy's days were very few without immediate relief, and so informed the parents. I explained to them what I must do, with their consent, to which they reluctantly agreed. On the 8th day of May I introduced an exploring trocar and canula between the fifth and sixth ribs, directly below the nipple, to ascertain whether I would certainly get pus or serum, that I could tell with a certainty about the proper size of the orifice that I must make for the evacuation of the contents of the chest. While the canula was still in, I passed a common bistory immediately under the instrument into the cavity and drew off at once through my flattened silver canula, that I used in the first place, three and a half pints of thin virulent matter having a greenish tinge. I then introduced a small gum catheter and rinsed the cavity with warm water, after which I at once injected it with the solution of Iodine, drew it off and introduced a tent which I fastened down by means of adhesive strips to prevent the introduction of air into the cavity. On the 9th I removed the tent and drew off half a pint of pus, and repeated the rinsing and injection of Iodine in the cavity as the day before, and so continued the same process for near one week. At the end of this time finding that the quantity of pus was not materially diminishing from day to day, as I could have desired, I determined to use the solution of Sulph Zinc., as in case two, on every alternate day; and on the days that I used the solution of Iodine, after rinsing with warm water, I simply introduced two or three ounces of the solution through a gum catheter and let it remain in the cavity. This I followed up for a couple of weeks, the discharge diminishing slowly and steadily. In the mean time I had taught the father to attend to the operation himself, so that I only visited him once per week leaving directions for the cavity to be evacuated every day, and washed out as usual, and the solution of Iodine to be used once in three or four days. The capacity of the cavity began to be lessened, and grew smaller and smaller till the 1st day of Aug. 1864. I closed the orifice and it speedily healed. I gave him, from the commencement, Opium Quinine, and Mur. Tinct. of Iron, and compelled him to take animal broths as a common drink. Three times a day he was allowed a roast potato, or a baked apple, and sometimes a little bread. Under this treatment the boy rapidly gained his strength. His chest is very little deformed, and now at this time he is hale and hearty and works steadily on the farm, making no complaint of any uneasiness about the chest.

Case 4—Would simply be a rehash of what I have before said in the preceding cases, and there being nothing particular in the case, it would be useless to report it in this place.

The only noticeable differences in the treatment of the foregoing cases, from that usually pursued when an operation has to be resorted to, is the more frequent evacuation of the cavity of its contents, giving the lung a better chance to recover from the pressure in the air cells that the quantity of pus must necessarily produce, preventing a free admission of air as well as blood, thereby interfering with the nutrition of the lung as well as the oxydation of the blood. From what I have observed in these cases, it is also my opinion that the admission of air into the pleural cavity is not attended with such evil consequences as we have heretofore been led to believe; the contact of the air with the puss being the principal evil, rendering putrifaction more speedy.

From my little experience in these cases I would not at all hesitate to introduce this solution of Iodine into the pleural cavity in case of Hydrothorax, and would expect by so doing, to change the condition of the secreting surface so as not only to lessen the amount of fluid secreted, but also to increase the power of absorption, thus balancing the two functions by restoring their equilibrium, and consequently establishing a healthy action.

